# Arsenic Exposure and the Induction of Human Cancers

**DOI:** 10.1155/2011/431287

**Published:** 2011-11-15

**Authors:** Victor D. Martinez, Emily A. Vucic, Daiana D. Becker-Santos, Lionel Gil, Wan L. Lam

**Affiliations:** ^1^Department of Integrative Oncology, British Columbia Cancer Research Centre, 675 West 10th Avenue, Vancouver, BC, Canada V5Z 1L3; ^2^Biomedical Sciences Institute, Faculty of Medicine, University of Chile, Independencia 1027, 8380453 Santiago, Chile

## Abstract

Arsenic is a metalloid, that is, considered to be a human carcinogen. Millions of individuals worldwide are chronically exposed through drinking water, with consequences ranging from acute toxicities to development of malignancies, such as skin and lung cancer. Despite well-known arsenic-related health effects, the molecular mechanisms involved are not fully understood; however, the arsenic biotransformation process, which includes methylation changes, is thought to play a key role. This paper explores the relationship of arsenic exposure with cancer development and summarizes current knowledge of the potential mechanisms that may contribute to the neoplastic processes observed in arsenic exposed human populations.

## 1. Introduction

Arsenic (As) is a chemical element classified as a metalloid. The most common oxidation states in the environment are +3 (As^III^, also known as arsenite) and +5 (As^V^ or arsenate), which exhibit different grades of toxicity [1]. Arsenic compounds can be found in organic (when linked with carbon and hydrogen) and inorganic (when combined with oxygen, chlorine, and sulfur, among other elements) forms [2]. 

Long term ingestion of inorganic arsenic has been associated with several human diseases. There are various sources of ingested arsenic, such as food (mainly in fish and seafood, algae, and cereals), air (coal-fired power generation and smelting), and water [3]. Of the various sources of arsenic in the environment, long-term exposure of arsenic in drinking water likely poses the greatest threat to human health [4]. Given its daily and widespread consumption, occurrence of arsenic in drinking water has been increasingly recognized as a major public health concern in several regions of the world over the past decades [5–7]. In fact, groundwater used for drinking contaminated with naturally occurring inorganic arsenic in Bangladesh represents one of the largest mass poisoning of a population in history [8]. Worldwide, an estimated 160 million people live in regions with naturally elevated levels of arsenic in drinking water, due to the presence of arsenic-rich geological formations [7].

Arsenic is a natural component of rocks containing copper or lead, which can result in release of arsenic into water or air in zones of intensive mining activities [6]. Due to these geological conditions and/or anthropogenic activities, soil and water supplies in these areas contain high concentrations of arsenical compounds [9]. This situation is compounded in extremely arid zones (such as in Northern Chile), where water sources are scarce and contaminated water serves as a drinking and irrigation supply. This can lead to massive chronic poisoning—called arsenicosis—affecting local populations [10]. Arsenic-contaminated drinking water represents an important public health issue, especially for developing countries.

Due to its physical characteristics (no odor, no color, and no flavor), arsenic exposure is often unnoticed, especially when ingested through drinking water. In this context, long-term effects are a major health concern in affected areas. The World Health Organization (WHO) and the U.S. Environmental Protection Agency have recommended a threshold of 10 *μ*g/L for inorganic arsenic concentration in drinking water [11, 12]. Unfortunately, millions of people are exposed to toxic levels and are at increased risk for the adverse health effects of arsenic [6, 13]. Concentrations exceeding this threshold have been described in Bangladesh, India, China, Argentina, Mexico, Canada, USA, and Chile, among other countries [14].

There is a strong body of evidence linking arsenic with a variety of health problems, from acute toxicities to chronic diseases which can take years to develop. Arsenic-related diseases include skin lesions, hypertension, ischemia, some endemic peripheral vascular disorders (e.g., “black foot disease”), diabetes, severe arteriosclerosis, neuropathies, and, significantly, many types of cancer [15–18]. Cancer-death risk associated with daily consumption of 1.6 liters of water with inorganic arsenic (50 *μ*g/L) has been estimated to be 21/1,000 [19].

Arsenic has been classified as a class I human carcinogen by the International Agency of Research on Cancer (IARC), meaning that there is sufficient evidence of carcinogenicity to humans. Despite evidence in humans, animal models fail to replicate these observed effects, hampering elucidation of the exact mode(s) of action underlying arsenic related carcinogenicity [20]. Skin and several types of internal cancers, including bladder, kidney, liver, prostate, and lung have been associated with arsenic ingestion [10, 21–25]. Skin cancer is the most common form of neoplasm associated with arsenic ingestion, while lung cancer corresponds to the most deadly [13, 26]. Interestingly, arsenic (specifically arsenic trioxide or As_2_O_3_) has been used as a chemotherapeutic agent for several types of cancer, with some studies showing high percentage of response in patients with acute promyelocytic leukemia (APL) [27, 28]. We will also discuss this issue in further sections.

## 2. Common Arsenic-Induced Malignancies

### 2.1. Skin Cancer

The relationship between arsenic and skin cancer has been well documented over the past several decades [29, 30]. The first inferences were made through observations of an increased frequency of skin cancer cases following treatment with Fowler's solution (1% potassium arsenite), formerly used for a variety of skin and hematological disorders [31]. Bowen's disease (intraepithelial carcinoma or carcinoma *in situ*), basal cell carcinoma (BCC)- and squamous cell carcinoma (SqCC) are the most common malignancies found in patients with long-term exposure to arsenic. Merkel cell carcinoma, an uncommon and highly aggressive cutaneous neoplasm, has been also documented at a lower frequency [32–35].

Arsenic-related skin SqCC can develop either *de novo* or progress from Bowen's disease, whereas arsenic-related BCC develops usually in multiple foci and areas of the body covered from sun exposure, in contrast to cases originating from other skin carcinogens, such as UV-light [36–38]. Arsenic-related Bowen's disease can appear 10 years after arsenic exposure, while other types of skin cancer can have a latency period of 20 or 30 years [39]. A dose-response relationship and cell-type specificity have been described for arsenic-related skin cancer [40, 41]. Additionally, normal human epidermal keratinocytes exposed to varying noncytotoxic/slightly cytotoxic concentrations of inorganic arsenic exhibit gene expression changes associated with molecular pathways relevant to arsenic-related skin carcinogenesis, such as oxidative stress, increased transcriptional levels of keratinocyte growth factors, and modulation of MAPK and NF-*κ*B pathways [42].

Premalignant skin lesions are relatively early manifestations of arsenic toxicity and are often considered precursors to arsenic-induced skin BCC and SqCC tumors [43]. These lesions include dermal manifestations such hyperpigmentation (a finely freckled, “raindrop” pattern of pigmentation or depigmentation) and hyperkeratosis (skin thickening, mainly at palms and the feet). These lesions are commonly found in chronically exposed populations and are considered a diagnostic criterion of arsenicosis [44]. Moreover, some genetic susceptibilities to these arsenic-related skin lesions have been proposed, since they do not occur in every exposed individual [45]. Hyperkeratosis can appear with shorter periods of arsenic exposure, and it has been described that these lesions give rise to the majority of arsenic-induced skin cancer [46, 47]. Additionally, it has been demonstrated that a significant proportion of fatal cases of skin cancer occurred in patients with prior signs of arsenicosis, such as keratosis and hyperpigmentation [31, 48].

In addition to directly affecting the carcinogenic process, it has been demonstrated that arsenic toxicity can also be potentiated by other environmental carcinogens. For example, arsenic-exposed individuals with a history of smoking and chronic exposure to environments with high fertilizer use may be more susceptible to cancer-prone skin lesions than those without these risk factors, even at the same level of arsenic exposure [43]. Arsenic can act as a cocarcinogen with UV light in a synergistic mode of action, leading to development of hyperkeratosis [49, 50]. Additionally, the same mode of action was observed between high levels of arsenic (over 100 *μ*g/L) and tobacco smoking with respect to risk of skin lesions in men [51].

### 2.2. Lung Cancer

There exists a significant dose-response relationship between arsenic concentration in water and incidence of lung cancer and other malignancies for both men and women [52]. The association between lung cancer and ingested arsenic was discovered following therapeutic application of this metalloid in psoriasis patients treated with Fowler's solution [53–55]. Thereafter, an increased lung cancer risk following exposure to arsenic in drinking water was demonstrated by several case-control and cohort-type studies [20]. Consistent, positive, and statistically significant associations among individuals exposed to high concentrations of arsenic in drinking water and increased risk of lung cancer have been detected [56, 57]. Based on large epidemiology studies in 1999, a report from the National Research Council (NRC, USA) concluded that there was sufficient evidence suggesting that the ingestion of arsenic in drinking water causes lung cancer, among other types of malignant neoplasias [58]. After this publication, other major arsenic and lung cancer epidemiological studies were published [25, 59]. Due to mounting evidence, the NRC study was reevaluated in 2001, concluding that the carcinogenic effects of arsenic in humans are significant, and that lung (and bladder) cancer should continue to be the focus of arsenic risk assessment for regulatory decision making [60].

 Interestingly, the increase risk of lung cancer associated with arsenic seems to be cancer subtype specific. For example, where SqCC incidence had decreased worldwide and overwhelmingly associated with cigarette smoking; in Northern Chile, a high proportion of SqCC frequently occurs in never smokers who have been chronically exposed to arsenic [25, 61].

As mentioned, Bangladesh represents the largest mass poisoning of a population in history, as groundwater used for drinking is contaminated with naturally occurring inorganic arsenic [8]. In rural areas in Bangladesh, arsenic contamination in drinking water from tube wells is associated with lung cancer in males, with lung SqCC being the predominant histological subtype in areas with arsenic concentrations above 100 *μ*g/L [62]. In these areas, the lifetime mortality risks of lung cancer are 159.1/100 000 for males and 23.1 for females (per 100 000 population) [63]. 

Blackfoot disease (BFD) is an endemic, peripheral arterial disease characterized by severe systemic arteriosclerosis and spontaneous gangrene resulting in amputation, common to individuals exposed to arsenic in Southwestern Taiwan [18]. In zones affected by BFD, increased incidence and subsequent mortality rates for lung cancer have been demonstrated, especially among those who used arsenic-contaminated well water for ≥40 years [64]. Smokers in this area have a 4.1-fold higher relative risk for lung cancer, suggesting a possible synergistic relationship between arsenic and tobacco exposures in terms of lung tumorigenesis [65]. Also, short-term exposure (5 years) of arsenic-contaminated drinking water (≥0.05 *μ*g/L) can also result in elevated lung cancer risk [66].

The Andean zone in South America is another area where the relationship between chronic arsenic exposure and lung cancer has been demonstrated. A dose-response relationship between arsenic in drinking water and lung cancer was found in central regions of Argentina [67], where arsenic concentrations in water supplies were >100 *μ*g/L, even reaching as high as 2000 *μ*g/L [68]. A correlation between increased arsenic concentration in drinking water and lung cancer incidence was also discovered in Northern Chile [25]. In this area, lung cancer mortality increased ten years after the initiation of high-level exposure (arsenic concentration >90 *μ*g/L in 1958) [10].

Chronic exposures to water contaminated with low concentrations of arsenic do not, however, show the same strong associations with increased cancer incidence and mortality. For example, a study carried out in Denmark [69] did not find any significant association between exposure to low concentrations of arsenic in drinking water (0.05–25.3 *μ*g/L) and risk of melanoma or lung cancer, among other types of neoplasias. Similarly, another study conducted in Belgium did not find a significant correlation between exposure to drinking water containing relatively low arsenic concentrations (20–50 *μ*g/L) and lung cancer mortality [70].

**Figure 1:**
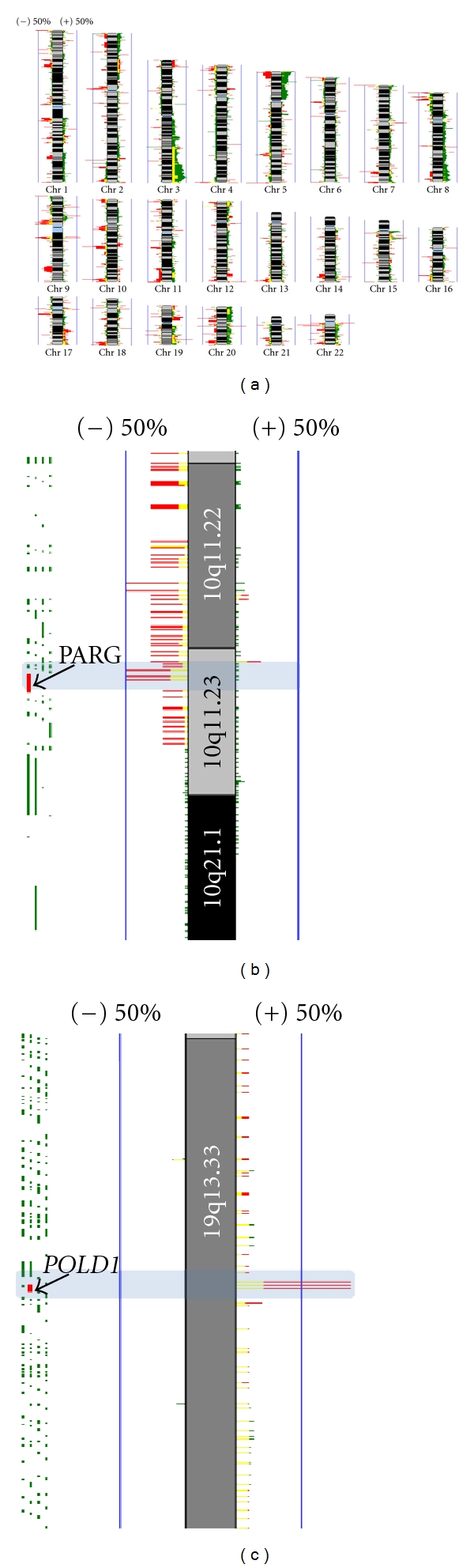
Genome-wide identification of arsenic-related and smoking independent DNA copy number alterations in lung squamous cell carcinoma (SqCC). Genomic copy number profiles for lung SqCC biopsies (*n* = 52) were obtained using whole genome aCGH. SqCC tumors from smokers (*n* = 42), comprised (*n* = 30) samples from North American with no known arsenic exposure, and (*n* = 12) samples from Northern Chile from individuals chronically exposed to arsenic. SqCC tumors from never smokers (*n* = 10) were from chronically arsenic-exposed individuals from Northern Chile. (a) Frequency plot of arsenic-related and smoking independent copy number differences in SqCC. The frequency of DNA gain/loss for each probe was calculated and plotted for each group, where smokers (dark green) and never smokers (red). Regions exhibiting similar alteration in both groups are denoted in yellow. The magnitude of green and red bars represents percent alteration for each probe per group (0–100%, with blue vertical lines representing 50% frequency). DNA gains and losses are represented to the right and left of each chromosome, respectively. Analysis was restricted to autosomes, with any differences based on sex subtracted from further analysis. A high frequency of copy number alteration, previously undescribed for SqCC were evident in arsenic exposed tumors from never smokers, particularly for chromosome 3q. (b) Detail of DNA losses at 10q11.23 specific to never smokers are highlighted in a light-blue rectangle. PARG, previously shown to mediate cell death in response to genotoxic stimuli (PMID: 19571039), is indicated in red. (c) Recurrent DNA gain found in never smokers at 19q13.33. This segment contains the *POLD1* gene, a DNA polymerase delta complex, involved in DNA replication and repair (red probe).

Genetic factors are thought to modulate susceptibility to arsenic-induced lung cancer. Carriers of CYP1A1*2A/GSTM1 homozygous deletion genotype show increased odds ratios for lung cancer, especially among smokers [71, 72]. In addition, our group has recently proposed that genomic aberrations in arsenic induced lung cancers exhibit distinct molecular characteristics. Using a whole genome tiling-path comparative genomic hybridization (CGH) array platform (described in [73]), we analyzed DNA copy-number alterations (CNAs) among lung SqCC cases from a Northern Chilean population chronically exposed to inorganic arsenic in drinking water (Figure 1) [74]. We identified unique patterns of chromosomal disruption and gene dosage related to SqCC from never smokers in Northern Chile, which did not correlate with normal DNA copy-number variations (Figure 1). This has led to the growing hypothesis that lung SqCC in arsenic-exposed individuals could represent a molecularly distinct form of this disease [74].

## 3. Carcinogenic Mechanisms of Arsenic Exposure

### 3.1. Arsenic Biotransformation as a Toxicity Activation Mechanism

Arsenic metabolism implicates a series of reduction and oxidation reactions. Pentavalent arsenical species are reduced to trivalent species, and oxidative methylation occurs to yield methylated tri- and pentavalent metabolites [75]. However, more than a detoxification mechanism, it has been proposed that methylation can activate the toxic and carcinogenic potential of arsenic, since it has been demonstrated that mono/dimethylated arsenical species (both tri/pentavalent) can affect gene transcription, and are more potent enzyme inhibitors and cytotoxins than non-methylated species [76, 77]. Moreover, since the arsenic biotransformation pathway uses S-adenosylmethionine (SAM) as a methyl group donor, arsenic can also interfere with a number of cellular processes that require methyl groups, leading to the idea that alteration of epigenetic mechanisms can also participate in arsenic-induced carcinogenesis [78]. A variety of these arsenic associated toxic events have been elucidated in cell line and animal models (Table 1). 

After reduction of As^V^ to As^III^ by purine nucleoside phosphorylase, As^III^ is methylated via a As^III^-methyltransferase, using SAM as a methyl group donor [79], producing mono and dimethylated trivalent species, such as monomethylarsonous acid (MMA^III^), dimethylarsinous acid (DMA^III^), and equivalent pentavalent species (monomethylarsonic acid or MMA^V^, and dimethylarsinic acid or DMA^V^. Interestingly, there is little evidence of methylated arsenic metabolites in skin, and *in vitro* studies have demonstrated that keratinocytes display very slow rates of arsenic methylation, and only mono-methylated species are produced [45, 80]. It has been proposed that As^III^ could be one of the responsible agents in arsenic related skin carcinogenicity, since it acts as a cocarcinogenic to mouse skin [81]. Additionally, individuals with arsenic-related skin lesions or skin cancer exhibit lower levels of dimethylated species in urine (in contrast to monomethylated species) [82–85], indicating that a lower methylation activity could predispose individuals to arsenic-related skin malignancies.

### 3.2. Arsenic-Induced Oxidative Stress 

Cellular induced damage derived from arsenic biotransformation leading to carcinogenic processes have usually been described to occur through oxidative stress by generation of toxic species, such as reactive oxygen species (ROS) leading to genomic aberrations. Generation of ROS has been described as one of the earliest and most important mechanisms of arsenic-induced carcinogenicity [86–91]. Oxidative damage (measured as guanine oxidation) is significantly associated with skin tumors associated with arsenic exposure [92, 93]. It has also been shown that oxidative stress can modify gene transcription profiles of human hyperkeratosis, affecting several cancer-relevant pathways, such as the Wnt/*β*-catenin and calcium signaling pathways [94, 95]. Both single- and double DNA strand breaks are characteristic of most cancer types and have been shown to be induced by chronic arsenic exposure, even at low concentrations [96, 97].

### 3.3. Epigenetic Changes

**Figure 2:**
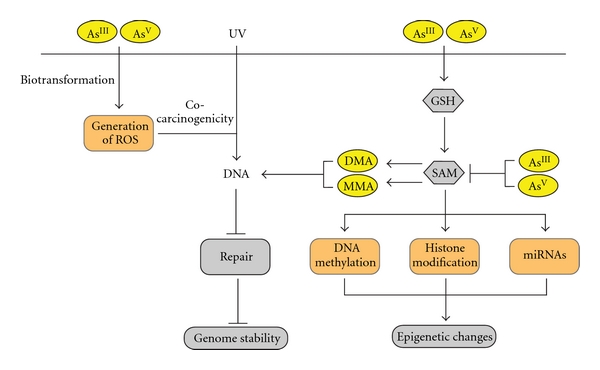
Schematic representation of proposed arsenic-induced carcinogenic mechanisms. Arsenic can enter cells in both tri- or pentavalent forms (As^III^ or As^V^). Inside cells, As^V^ is converted to As^III^, with subsequent methylation to monomethylated (MMA) and dimethylated (DMA) species. The methylation of inorganic arsenic consumes both S-adenosylmethionine (SAM) and glutathione (GSH). Cellular damage derived from arsenic biotransformation can occur through generation of reactive oxygen species (ROS), and through epigenetic mechanisms: changes in DNA methylation patterns (by depletion of cellular pools of methyl group), histone modification, and altered expression of microRNAs (miRNAs).

Arsenic, arsenic metabolites, and metabolism, directly and indirectly, affect normal epigenetic transcriptional regulation at both the level of DNA methylation, histone maintenance, and miRNA expression (reviewed in [98, 99]). As previously mentioned, biotransformation and reduction of arsenic leads to the formation of highly toxic methylated arsenic species which act as potent cytotoxics and enzyme inhibitors. Since this process utilizes SAM, the cell's own methyl group donor, arsenic is thought to interfere with the cell's ability to maintain normal epigenetic regulation via the disruption of normal DNA methylation patterns, histone modification, and expression of microRNAs (miRNAs), possibly by the depletion of cellular pools of methyl groups (Figure 2). Epigenetic modifications do not alter the DNA sequence itself but instead result in chemical modifications to DNA or histone tail residues. Cells and tissues exposed to arsenic display epigenetic aberrations that mimic early hallmarks of cancer, providing evidence for an epigenetic role in arsenic-mediated tumorigenesis. Epigenetic regulation of gene expression is a highly dynamic process that can be modulated by existing therapeutics [100, 101] which may potentially apply to arsenic-related malignancies.

#### 3.3.1. DNA Methylation

In the human genome, DNA methylation occurs at the 5-carbon position of cytosine in CpG dinucleotide sequences, resulting in 5′-methylcytosine (5mC), often within short evolutionarily conserved regions enriched for CpG dinucleotides, called CpG islands [102, 103]. When located in promoter regions of genes, CpG islands are typically unmethylated (~90%); however, promoters without CpG islands are frequently methylated [104]. Therefore, the bulk of methylated DNA in the human genome occurs in repetitive DNA sequences, where it is thought to have an important role in silencing transposable elements and maintaining genomic stability. The transfer of the donor methyl group from SAM to the cytosine in a CpG dinucleotides is catalyzed by DNA methyl transferase (DNMT) enzymes, responsible for the *de novo* methylation throughout development (DNMT3a, DNMT3b, and DNMT3L) and maintenance of methylation patterns in somatic tissue (DNMT1). Aberrant DNA methylation is implicated in a vast spectrum of diseases and disorders and is one of the earliest and most frequent aberrations in cancer. DNA hypomethylation is associated with genomic instability and the reexpression of parasitic DNA, in addition to activation of genes normally silenced by methylation in a tissue-specific manner. Conversely, aberrant DNA promoter hypermethylation is strongly linked to transcriptional gene silencing, particularly for tumor suppressor genes (TSGs) and cancer.

DNA methylation patterns observed in human cancers resemble those of arsenic-related premalignant and malignant cells and tissues. For example, the DNA methylation patterns of several well-known cancer genes have been studied in the context of, and found to correlate with, arsenic exposure in *in vitro *cell models and in populations exposed to arsenic. The activation of proliferative genes in rat liver epithelial cell lines which undergo malignant transformation following chronic, low-level arsenic exposure is associated with aberrant DNA hypomethylation [105]. Exposure to As^III^ and As^V^ in the lung cancer cell line (A549), resulted in promoter hypermethylation and subsequent transcriptional silencing of *p53* [106]. Mice, chronically exposed to As^V^ through drinking water, acquire frequent promoter hypermethylation of the tumor suppressors *p16^INK4A^* and *RASSF1A* in lung tumor tissues [107]. A study analyzing 351 cases of bladder cancer cases found that arsenic exposure was associated with promoter methylation of *RASSF1A* and *PRSS3* [108]. Intriguingly, in one study, individuals with no cancer, but who were exposed to high arsenic concentrations (>251 *μ*g/L), had a significant degree of DNA hypermethylation in promoter regions of *p53* and *p16^INK4A^* compared to nonexposed controls [109], suggesting that arsenic related-hypermethylation events may be some of the earliest causal tumorigenic events. Collectively, these and other findings (see [98, 99]) support the notion that arsenic-induced changes to DNA methylation play a role in tumor formation. 

#### 3.3.2. Histone Modification

Histones proteins enable condensation of double-stranded supercoiled eukaryotic DNA into nucleosomes, which are made up of two copies each of H2A, H2B, H3, and H4 proteins. The N-terminal tails of histones are accessible to modifying enzymes, which function in catalyzing posttranslational modifications to the amino acid residues residing within the histone tail, including acetylation, methylation, ubiquitination, sumoylation, and phosphorylation amongst others [110, 111]. Specific patterns of these modifications, commonly referred to as the “histone code”, correlate with transcriptional states of associated genes as well as to disease phenotypes. Working in conjunction with transcriptional coactivators or repressors, histone modifying enzymes catalyze the addition or removal of these modifications to generally induce or maintain an (1) open euchromatic state, through the addition of acetyl groups (via histone acetyltransferases) or (2) a closed or heterchromatic state, through the addition of methyl groups (via histone methyltransferases) or removal of acetyl groups (via histone deacetylases) on specific histone residues. Therefore, transcriptional activity of associated genes correlates with the formation of euchromatin or heterochromatin. 

Arsenic metabolites have been shown to modulate normal histone patterns. As^III^ has also been shown to modify methylation patterns on H3K4, H3K9, and H3K27 [112]. A549 cells exposed to 2.5–5 *μ*M of As^III^ exhibited an increase in H3K9 dimethylation and a decrease in H3K27 trimethylation, both of which are associated with heterochromatin (gene silencing), and a decrease in H3K4 trimethylation which is associated with euchromatin formation (an activation mark). When the normal bronchial epithelial cell line (BEAS-2B) was exposed to 1-2 *μ*M of As^III^, an increase in dimethylation of H3K9 was observed.

Arsenic compounds were also shown to induce malignant transformation of human nontumorigenic cell lines through changes to histone H3 acetylation, DNA promoter methylation, and decreases expression of the *DBC1*, *FAM83A*, *ZSCAN12*, and *C1QTNF6* genes [113]. For each of these underexpressed genes, DNA methylation inversely correlated with the histone acetylation levels for their respective promoter regions, leading authors to conclude that changes in histone H3 acetylation occur during arsenic-induced malignant transformation.

#### 3.3.3. MicroRNAs

miRNAs are small, noncoding RNA species that orchestrate the expression of genes involved in many key aspects of cell biology by degradation and translational inhibition of their target mRNAs (reviewed in [114]). In humans, more than 1400 miRNAs have been identified to date (miRBase data base; Release 17, April 2011). miRNAs inhibit gene expression by binding to the 3′-untranslated region of mRNAs through imperfect base pairing; consequently, a single miRNA can negatively regulate the expression of multiple and sometimes upwards of hundreds target genes. As a result, miRNAs deregulations are implicated in diverse human pathologies, including cancer (reviewed in [115]).

An increasing number of studies show that arsenic exposure can alter miRNA expression levels *in vitro* and *in vivo. *Human lymphoblastoid cells exposed to sodium As^III^ over six days showed altered expression of five miRNAs (hsa-miR-210, -22, -34a, -221, and -222) [116]. The authors hypothesized that these alterations could be a consequence of changes in methylation patterns, since the same alterations were observed when cells were grown under folate-deficient conditions, which can lead to reduced levels of SAM. Furthermore, overexpression of hsa-miR-222 was confirmed in human peripheral blood-derived cells from individuals with insufficient dietary folate. The induced changes in miRNA expression could be reversed by the restoration of folate, suggesting that continuous exposure to agents like arsenic may be necessary to permanently alter the expression of miRNAs.

Chronic exposure to As^III^ has also been shown to induce malignant transformation and epithelial-to-mesenchymal transition (EMT), in concert with reduction in levels of miR-200 family members in immortalized p53-knocked downhuman bronchial epithelial cells (HBECs) but not in p53-intact HBECs [117]. Interestingly, stable expression of miR-200b alone was capable of entirely reversing and preventing As^III^-induced EMT and malignant transformation. Arsenic exposure depleted the miR-200 s through the induction of EMT-inducing transcription factors zinc-finger E-box-binding homeobox factor 1 (ZEB1) and ZEB2 and increased methylation of miR-200 promoters. 

A recent study examining the global expression of miRNAs and mRNAs of chick embryos after arsenic exposure revealed a dramatic decrease in expression of miRNA-9, -181b, -124, and -125b [118]. NRP1—a transmembrane receptor involved in angiogenesis—which is upregulated at the mRNA level in arsenic-treated chick embryos was found to be a target gene of miR-9 and miR-181b. Overexpression of miR-9 or miR-181b suppressed As^III^-induced NRP1 expression, cell migration and tube formation, supporting involvement of these miRNA species in As^III^-induced angiogenesis via NRP1 gene activation.

Despite its carcinogenic potential, As^III^ has also been used as a treatment option for APL, which is frequently associated with a gene fusion involving the retinoic acid receptor alpha (RARA) and the promyelocytic leukemia protein (PML) gene [119]. Saumet et al. have shown that PML-RARA is able to transcriptionally repress several miRNAs associated with critical pathways linked to leukemogenesis, such as HOX proteins and cell adhesion molecules [120]. Expression of these miRNAs was restored by retinoic acid and As^III^, suggesting that, in APL, these agents may function to inhibit cell growth, at least in part by impacting miRNA expression.

## 4. Conclusion

Arsenic contamination of drinking water remains a serious public health problem, affecting hundreds of millions individuals worldwide. The more severe effects, such as cancer, are evident up to several decades after exposure has ceased. Although mitigation measures have been taken, the natural origin of this contamination keeps this problem an active preoccupation that requires strategies to monitor arsenic concentrations in drinking water and to define markers associated with early health effects.

Overall, reviewed literature indicates that arsenic exposure exerts deleterious health effects primarily through the induction of oxidative stress, alterations to DNA methylation, histone modification, and miRNA expression. Understanding these events in the context of arsenic toxicity may provide powerful biomarkers for arsenic-induced carcinogenicity and elucidation of early steps in arsenic-induced malignancies that may be reversed by targeted therapies or preventative chemotherapeutics. Larger, carefully designed epidemiologic studies will be required to more comprehensively examine the presence and consequence of these alterations in populations affected by arsenic contamination. Since synergistic cocarcinogenicity, especially in skin and lung cancer, occurs in arsenic-exposed individuals, considerations of other environmental agents should be taken into account in these studies. Elucidation of the mechanisms underlying the initiation and promotion of carcinogenesis related to arsenic's biotransformation processes and metabolites is of foremost importance to the development of early detection and treatment regimes for affected individuals.

## Figures and Tables

**Table 1:** Changes in functions associated to arsenic-related carcinogenicity.

Type of alteration	Cell model/type	As species	Reference
*Associated to oxidative stress*			
DNA strand break	Human fetal lung fibroblast (2BS cells)	As^III^	[121]
DNA strand break	Human alveolar epithelial type II (L-132) cells	DMA^V^	[122]
Single-strand DNA breaks, DNA-protein adducts, sister chromatid exchanges	Human fibroblast cell lines	As^III^	[123]
Formation of apurinic/apyrimidinic sites	Human alveolar epithelial cell line (L-132)	DMA	[124]
Induction of 8-OHdG	Human breast cancer MCF-7 adenocarcinoma epithelial cells	As^III^	[125]
Increases 8-oxo-G levels through (CH3)_2_AsOO		DMA^V^	[126, 127]
Presence markers for oxidative stress were detected, including 8-oxodG	Mouse bronchiolar Clara cells	DMA^V^	[128]
Double-strand DNA breaks	Mammalian cells		[96]

*Epigenetic changes in DNA methylation/histones modification/miRNA expression*			
Alteration of methylation in p53 promoter	A549 cell line	As^III^ As^V^	[106]
Inductor of hypermethylation of the p16INK4a and RASSF1A CpG islands in nontumor lung tissues (including hyperplasia and adenoma) and lung adenocarcinomas	Lungs of mice exposed during 18 months	As^V^	[107]
Increase of dimethylated H3K9	Human BEAS-2B cell line	As^III^	[112]
Increased H3K9 dimethylation and decreased H3K27 tri-methylation (gene silencing), increasing H3K4 tri-methylation (gene-activating mark), increases histone methyltransferase G9a protein levels	Human A549 cell line	As^III^	[112]
Changes to histone H3 acetylation, DNA promoter methylation, and decreases expression of the *DBC1*, *FAM83A*, *ZSCAN12*, and *C1QTNF6* genes	Human nontumorogenic cell lines		[113]
Altered expression of hsa-miR-210, -22, -34a, -221, and -222	Human lymphoblastoid cells	As^III^	[116]
Reduction in levels of miR-200	Immortalized p53-knocked down human bronchial epithelial cells (HBECs)	As^III^	[117]
Decrease in expression of miRNA-9, -181b, -124, and -125b	Chick embryos	As^III^	[118]

*Other changes*			
Amplification of the dihydrofolate reductase gene	Mouse 3T6 cells	As^III^	[129]
MAPK activation; phosphorylation of ATF-2 and c-Jun, elevated IL-8 release	Human BEAS 2B line	As^III^	[130]
Induction of p53-independent expression of GADD45 protein (a G2/M cell-cycle checkpoint protein)	Human BEAS 2B line	As^III^	[131]
Stabilization of GADD45 alpha mRNA through nucleolin	Human BEAS 2B line	As^III^	[132]
Mostly decreased expression for transcripts involved in angiogenesis, lipid metabolism, oxygen transport, apoptosis, cell cycle, and immune response	Lung of mice exposed	As^III^	[133]
Induction of the expression of genes involved with cancer, the cell cycle, cellular proliferation, DNA replication, recombination and repair, lipid metabolism, cell-cell signaling and interaction, molecular transport, and immunological disease pathways in Ogg1^−/−^ mice	Lungs of Ogg1^−/−^ mutant mice exposed	DMA^V^	[134]
Enhanced centrosome amplification in p53-compromised cells. Resistance to arsenite-induced G2/M cell cycle arrest and arsenite-induced apoptosis in p53-compromised cells. Reductions in arsenite-induced enhancement of p53, p21, and Gadd45a expressions (at 5–10 *μ*M), Higher (200%) cell colony formation in p53-inhibited BEAS-2B cells (5 *μ*M)	H1355 cells (human lung adenocarcinoma cell line with mutation in p53) Human BEAS-2B line p53-inhibited BEAS-2B cells	As^III^	[135]
Increased expression of ER-alpha and genes related to estrogen signaling in the fetal lung of female mice	Lung samples from gestation day 18 female fetal C3H mice	As^III^	[136]
Downregulation of (validated genes): Tpi1, Ldha, and Pgk1. Upregulation of (validated genes): Cox6a2; ^V^ariable: Id1, Gpnmb	Rat lung epithelial cell line (L2)	As^III^	[137]
Increased cell viability (≤0.5 *μ*M). Downregulation of APE1 and Pol*β* mRNA (above 1 *μ*M)	GM847-immortalized human lung fibroblast	As^III^	[138]
Increased plating efficiency (cell growth advantage), micronuclei incidence (marker of chromosomal instability), gene amplification (PALA resistance), invasive capabilities; anchorage-independent growth (oncogenic transformation); lost of *β*4 integrin expression; upregulated phosphorylation of Rb and ERK; decreased expression of p53 protein	h-TERT-immortalized human small airway epithelial cells	As^III^	[139]
